# A Multidisciplinary Approach Establishes a Link between Transglutaminase 2 and the Kv10.1 Voltage-Dependent K^+^ Channel in Breast Cancer

**DOI:** 10.3390/cancers15010178

**Published:** 2022-12-28

**Authors:** Rita Canella, Federica Brugnoli, Mariana Gallo, Jeffrey W. Keillor, Anna Terrazzan, Elena Ferrari, Silvia Grassilli, Eric W. J. Gates, Stefano Volinia, Valeria Bertagnolo, Nicoletta Bianchi, Carlo M. Bergamini

**Affiliations:** 1 Department of Neuroscience and Rehabilitation, University of Ferrara, 44121 Ferrara, Italy; 2 Department of Translational Medicine, University of Ferrara, 44121 Ferrara, Italy; 3 Department of Medicine and Surgery, University of Parma, 43125 Parma, Italy; 4 Department of Chemistry and Biomolecular Sciences, University of Ottawa, Ottawa, ON K1N 6N5, Canada

**Keywords:** breast cancer, MDA-MB-231 cells, transglutaminase 2, transglutaminase inhibitors, AA9, Kv10.1, *KCNH1*, K^+^ channel, patch-clamp

## Abstract

**Simple Summary:**

This work intends to unravel one of the roles played by transglutaminase 2 within the cell. We highlighted its physical interaction with the voltage-dependent Kv10.1 K^+^ channel, an important target of therapies in breast cancer. The use of transglutaminase 2 inhibitors can selectively affect the membrane current of triple-negative cells in which this channel is functional.

**Abstract:**

Since the multifunctionality of transglutaminase 2 (TG2) includes extra- and intracellular functions, we investigated the effects of intracellular administration of TG2 inhibitors in three breast cancer cell lines, MDA-MB-231, MDA-MB-436 and MDA-MB-468, which are representative of different triple-negative phenotypes, using a patch-clamp technique. The first cell line has a highly voltage-dependent a membrane current, which is low in the second and almost absent in the third one. While applying a voltage protocol to responsive single cells, injection of TG2 inhibitors triggered a significant decrease of the current in MDA-MB-231 that we attributed to voltage-dependent K^+^ channels using the specific inhibitors 4-aminopyridine and astemizole. Since the Kv10.1 channel plays a dominant role as a marker of cell migration and survival in breast cancer, we investigated its relationship with TG2 by immunoprecipitation. Our data reveal their physical interaction affects membrane currents in MDA-MB-231 but not in the less sensitive MDA-MB-436 cells. We further correlated the efficacy of TG2 inhibition with metabolic changes in the supernatants of treated cells, resulting in increased concentration of methyl- and dimethylamines, representing possible response markers. In conclusion, our findings highlight the interference of TG2 inhibitors with the Kv10.1 channel as a potential therapeutic tool depending on the specific features of cancer cells.

## 1. Introduction

The multifunctionality of the type 2 transglutaminase (TG2) includes several extra- and intracellular activities [1,2,3]; regarding interactions with membrane and extracellular matrix proteins [4,5,6,7,8]; GTPase functions [9,10], signaling cascades [11,12,13]; modification of cytoskeletal structures [14,15], of transcription factors and of structural chromatin proteins [16,17,18,19]; or interference with calcium homeostasis [20] and metabolism [21,22]. TG2 inhibitors have been described as suitable in the pathologies in which the enzyme displays pivotal biological functions [23,24,25,26,27,28,29,30], although it is difficult to discriminate and understand the specific biological effects. Focusing on breast cancer (BC), TG2 inhibition improves the activity of anticancer drugs in the control of cell proliferation and apoptosis [31,32], reverses drug resistance [33] and limits cell motility during epithelial-to-mesenchymal transition (EMT) [14]. We can summarize that these effects are mediated by two different structures of the enzyme, an open state, associated with transamidation reactions involved in extracellular matrix modifications and activated by Ca^2+^, and a closed state with intracellular functions, such as binding and hydrolyzing GTP, although even inside the cell, TG2 can catalyze transamidation in the presence of a high Ca^2+^ concentration [34]. Both activities can be blocked by specific inhibitors, such as NC9, and the same can be done in BC with a triple-negative phenotype, showing elevated levels of TG2 [31]. Some compounds, notably AA9, not only block the transamidation properties of TG2, but they also prevent the G-protein’s role carried out by the closed conformation [35]. 

In BC, TG2 interferes with several pathways of cell signaling [34], usually involving intracellular targets localized in lipid rafts zones of membrane in which the number of ion channels is high. For this reason, we studied the impacts of TG2 inhibitors on membrane currents. Recently, interference by TG2 contained in exosomes was reported in L-type calcium channels, localized on the pre- and postsynaptic nerve terminals in the central nervous system [36], but in this study we focused on intracellular actions which are targetable to obtain antitumor effects [34].

In the specific context of BC, investigations of ionic fluxes have a long history. Membrane currents are frequently altered, and depolarization preferentially affects more aggressive cells [37], as in the case of triple-negative phenotypes, which represent types tumors that are still difficult to cure. Alterations in ion homeostasis could be caused by changes in the extracellular matrix and tumor microenvironment that mediate processes of angiogenesis, vascularization, EMT and migration, as a prelude to metastatic dissemination [38]. These events are strongly associated with calcium, copper, potassium, sodium and chloride homeostasis, so that the use of channel blockers, chelators and ion administration can oppose cancer progression. Their clinical relevance has stimulated the investigation of molecules to regulate ion channels, such as steroidal sex hormones and vitamin D [39], and at present, a wide number of compounds of therapeutic interest can be employed to limit or control specific ion channels [38]. Overall, K^+^ channels have a leading role and represent good candidates as markers. They are easily druggable, owing to their position on the cell membrane [40]. The voltage-dependent Kv10.1 channel (also named ether à-go-go-1, Eag1) has been indicated as a good target for therapy of most human tumors [41], despite its similar structure to the Kv11.1 channel (human ether-à-go-go-related gene, hERG), which is relevant for cardiac physiology. Indeed, they belong to the same family and display similar affinity for almost all blockers [42]. 

A few lines of experimental evidence have been reported in support of the relationship between K^+^ ion flux and TG2, documenting its involvement in the activation of the Ca^2+^-dependent Maxi-K^+^ channel of vascular smooth muscle cells, determined by the ability of TG2 to function as a G-protein [43]. We underline that cystamine inhibits TG2, affecting the phospholipase C pathway and leading to the opening of Kv channels, decreasing Ca^2+^ intracellular levels in rat mesenteric small arteries [44]. This has been demonstrated by the use of LDN 27219, which selectively blocks the closed form of the enzyme but not the transamidase function [25]. Other findings indicate a reverse relationship: blockers of K^+^ channels modify the Ca^2+^ concentration, interfering with the transamidating activity of TG2, as in keratinocyte differentiation [45], and the use of depolarising agents, such as veratridine or high potassium concentration, increased influx and accumulation of Ca^2+^ into rat ganglia, causing activation of TG2 catalysis [46]. 

In an attempt to unravel the intracellular functions of TG2, we have employed inhibitors that could affect not only its enzymatic transamidation, but also its G-protein activity [47]. Other than the well-known inhibitor NC9 [14,48,49], we used the novel compound AA9 [35]. We introduced these drugs into the cell by the patch-clamp pipette method to record the whole-membrane current [50]. The aim of this work is to answer the question: “Is there a connection between some kind of K^+^ channel and TG2 in aggressive BC?”. If so, the inhibitors of TG2 could help to control the K^+^ fluxes and tumor growth.

## 2. Materials and Methods

### 2.1. Cell Growth and Treatments

For our investigation we used three triple-negative BC cell lines with different characteristics, purchased from American Type Culture Collection (https://www.atcc.org/, accessed on 30 March 2022) (Rockville, MD, USA). MDA-MB-468 are epithelial cells of metastatic adenocarcinoma isolated from pleural effusion carrying mutations of the *PTEN*, *RB1*, *SMAD4* and *TP53* genes, which we grew in Dulbecco’s modified eagle’s medium (DMEM)/F-12 DMEM, Gibco Laboratories, New York, NY, USA), 10% fetal bovine serum (FBS, Gibco Laboratories, New York, NY, USA), 50 U/mL penicillin and 50 μg/mL streptomycin (Gibco Laboratories). The other two cell lines, with similar pleural effusion, were MDA-MB-231 cells with mesenchymal-like morphology presenting mutations in the *BRAF*, *CDKN2A*, *KRAS*, *NF2* and *TP53* genes, and the pleomorphic MDA-MB-436 cells with multinucleation and altered *BRCA, TP53* and *RB1* genes. They were cultured in DMEM High Glucose with stable L-Glutamine (EuroClone, Milan, Italy), 10% FBS and antibiotics. All cultures were grown at 37 °C with 5% CO_2_ in a humidified atmosphere.

Cell treatments were carried out with the addition of drugs to the culture medium, when the cells reached about 70% confluence. As TG2 inhibitors, we used the following compounds: N-α-carbobenzyloxy-N-ε-acryloyl-L-lysine(2-(2-dansylaminoethoxy)-ethoxy)ethanamide, also named NC9, and (S)-benzyl(1-(4-(1-naphthoyl)piperazin-1-yl)-6-acrylamido-1-oxohexan-2-yl)carbamate, called AA9, respectively, synthesized as described [46] and also commercially available from Zedira (Darmstadt, Germany). 

For the apoptosis assay the inhibitors were used at the final concentrations of 75, 50 and 25 μM; 0.1% DMSO was used as a negative control. 

In patch-clamp experiments, TG2 inhibitors were injected into a single cell: NC9 at the concentration of 3, 10 or 15 μM (for preliminary setting of the method), and AA9 at 9 μM; again, 0.1% DMSO was the negative control. The 4-aminopyridine (4-AP) (Merck Life Science, Milan, Italy), dissolved in water to achieve a final concentration of 5 mM, represented the positive control as a specific inhibitor of the K^+^ voltage-dependent channels [51,52]. In addition, we employed 10 μM astemizole (Merck Life Science, Milan, Italy), specifically to inhibit the Kv10.1 channel [53].

For metabolomic analysis, cells were grown in triplicate in T75 flasks (EuroClone), replacing the medium at 70% confluence. After 8 h of adaptation, we added the compounds to the culture and collected the cells after 16 h of exposure to 9 μM AA9 and 30 μM NC9 (in agreement with their K_I_ values), according to the procedure described in the literature [14]. Control cell cultures were grown in medium supplemented only with the solvent (0.1% DMSO). Each condition was performed in triplicate. Upon treatment, the cell-free medium of each flask was recovered and stored at −80 °C until sample preparation.

### 2.2. Detection of Apoptosis

Apoptosis was detected after 48 h of treatment with TG2 inhibitors using the Muse® Annexin V & Dead Cell Kit (Luminex, Prodotti Gianni, Milan, Italy) [31]. 

We performed an apoptotic assay on BC cells at 10^5^/well for the final concentration. The adherent cells were exposed to the action of trypsin for 2 min at 37 °C that was inactivated by using 1 mL of culture medium containing 10% FBS. The cells were centrifuged at 1200 rpm for 5 min, washed with fresh complete culture medium and 100 μL of cells were diluted with an equal volume of kit solution. We analyzed the cells by Muse after an incubation of 20 min.

Statistical analysis of apoptosis data was obtained by calculating *p* value by Student’s *t*-test or one-tailed homovariance. Significance is expressed by (*) *p* value < 0.05, (**) *p* value < 0.01, or (***) *p* value < 0.001. 

### 2.3. Quantification of Gene Expression by RT-qPCR 

Total RNA from 3 × 10^6^ cells was extracted by TRI Reagent®, as indicated in the manufacturer’s protocol (Merck Life Science). Reverse transcription (RT) was performed on 1 µg of total RNA using TaqMan® Reverse Transcription Reagents kit (ThermoFisher Scientific, Monza MB, Italy). To analyze TG2 gene expression, we carried out quantitative polymerase chain reactions (qPCRs) as described by Franzese et al. [54], using β-actin (ACT) as a reference gene, forward primer 5′-CATTGCCGACAGGATGCA-3′ and reverse primer 5′-GCTGATCCACATCTGCTGGA-3′. Fold change was determined by comparing the relative threshold cycle value (CT) of the target gene with that of the reference to obtain the ΔCT value. To quantify the increase/decrease, we applied the formula 2^−ΔCT^ expressing target modulation.

### 2.4. Patch-Clamp Technique

Patch pipettes were pulled from glass capillaries with a 1.0 mm outer diameter using a micropipette puller (NARISHIGE Instruments, Tokyo, Japan, mod PP-830), fire-polished (tip resistance between 2 and 5 MOhm) and filled with an intracellular solution. 

In order to characterize the overall membrane current response, we used an intracellular solution containing 145 mM KCl, 1 mM MgCl_2_, 10 mM HEPES and 5 mM EGTA, whose pH was adjusted with KOH to the value of 7.3; and an extracellular solution containing 145 mM NaCl, 1.8 mM CaCl2, 1 mM MgCl_2_, 5.4 mM KCl, 10 mM glucose, and 10 mM HEPES, whose pH was adjusted with NaOH up to the value of 7.35. If required, osmolality was adjusted with sucrose to obtain values between 300 and 310 mOsm/kg.

The 4-AP and astemizole were diluted at the time of use and added to the perfusion solution. NC9 and AA9 were added to the intracellular solution. We underline that TG2 inhibitors affected the adhesion of the pipette if administrated outside the cell during the patch-clamp, hampering any record.

Cells were viewed through a TV monitor connected to a contrast-enhanced video camera (T.I.L.L. Photonics, Planegg, Germany), coupled to an inverted microscope (Olympus IMT-2, Tokyo, Japan) equipped with a 40x Hoffman-modulation contrast objective.

Whole cell currents were elicited by voltage-clamp pulses (1400 ms duration) between +70 and −90 mV in 20 mV steps beginning from a holding potential of −30 mV (considered close to the membrane potential of non-excitable cells) [55,56].

The voltage protocol and data acquisition were performed with Digidata card 1322A and pClamp package (version 9.2, Molecular Devices, LLC. 3860 N. First Street, San Jose, California, 95134, USA). The currents were recorded with a commercial patch-clamp amplifier (EPC-7; Consumer E-List, Darmstadt, Germany); the recordings were filtered at 5 kHz, acquired at 10 kHz and stored on a disk.

Data from patch-clamp experiments are reported in the text and figures as mean ± SEM. Comparisons control to treatment were made with GraphPad Prism v.5 by two-way ANOVA and were considered significant for *p* values less than 0.05.

### 2.5. Immunoprecipitation and Immunochemical Analysis

Lysates from all cell lines were analyzed by Western blot after the addition of Laemmli’s SDS sample buffer to cells washed with cold phosphate buffer saline containing 1 mmol/L Na_3_VO_4_.

For immunoprecipitation experiments, cells were lysed, treated with protease and phosphatase inhibitors and incubated with antibodies against TG2 (Ig036, Zedira, Darmstadt, Germany), Kv10.1 (sc-398585, Santa Cruz Biotechnology, Santa Cruz, CA, USA) and Kv11.1 (sc-377388, Santa Cruz Biotechnology) or with a non-specific IgG, which was used as the negative control (sc-53344, Santa Cruz Biotechnology). They were immunoprecipitated with protein A-sepharose (Pharmacia, Uppsala, Sweden), according to the previously reported experimental conditions [14].

Total cell lysates and immunoprecipitates were separated on 7.5% denaturing polyacrylamide gel and blotted on nitrocellulose membranes (GE Healthcare Life Science, Little Chalfont, UK) and finally hybridized with antibodies against TG2, Kv10.1 and Kv11.1 and β-tubulin (T4026, Merck Life Science), as previously described [14,42,57].

The chemiluminescence of the resolved bands was acquired with an ImageQuantTM LAS 4000 biomolecular imager (GE Healthcare Life Science), and densitometrical analysis was performed by Image Quant TL software (GE Healthcare Life Science), as previously reported [58].

The results were expressed as means  ±  SDs of three independent experiments. Statistical analysis was performed by two-tailed Student’s *t* test for unpaired data using the GraphPad Prism 6.0 statistical package (GraphPad Software, San Diego, CA, USA). *p* values < 0.05 were considered statistically significant.

### 2.6. Samples’ Preparation for Metabolomic Analysis of Cell Supernatants 

For each triplicate sample, 1 mL of culture supernatant and of fresh medium were ultra-filtered using Amicon Ultra-4 Centrifugal filters (10,000 MWCO, Merck Millipore) at 4000× *g* and 5 °C to deplete the metabolite solutions of proteins. Sodium phosphate buffer (pH 7.4) and 3-trimethylsilyl propionic acid, used as a chemical shift reference (0.00 ppm) and quantitative internal standard, were added to 575 μL of the ultra-filtered solutions, reaching the final concentrations of 50 mM and 1.45 mM, respectively. Final samples also contained 2.5% D_2_O for the signal lock.

One-dimensional ^1^H-NMR spectra were acquired at 25 °C with a JEOL 600 MHz ECZ600R spectrometer (JEOL USA Inc., Peabody, MA, USA) according to Gallo et al. 2022 [59]. The spectra were processed and analyzed with the Chenomx NMR suite 9.0 software (Chenomx Inc., Edmonton, AB, Canada), zero-filling to 256 k points and processed using a line broadening of 0.5 Hz.

Multivariate statistical analysis was conducted on target metabolites using MetaboAnalyst 5.0 (https://www.metaboanalyst.ca, accessed on 25 May 2022) [60]. Metabolite concentrations were normalized to the median and auto-scaled before analysis. The heatmap analysis of the nine samples was performed using the Pearson distance measure and Ward clustering method. A volcano plot was employed for two-group data analysis, considering 2 and <0.01 as fold change (FC) and raw *p* value thresholds, respectively.

## 3. Results

### 3.1. Comparison between Apoptotic Effects of TG2 Inhibitors

**Figure 1:**
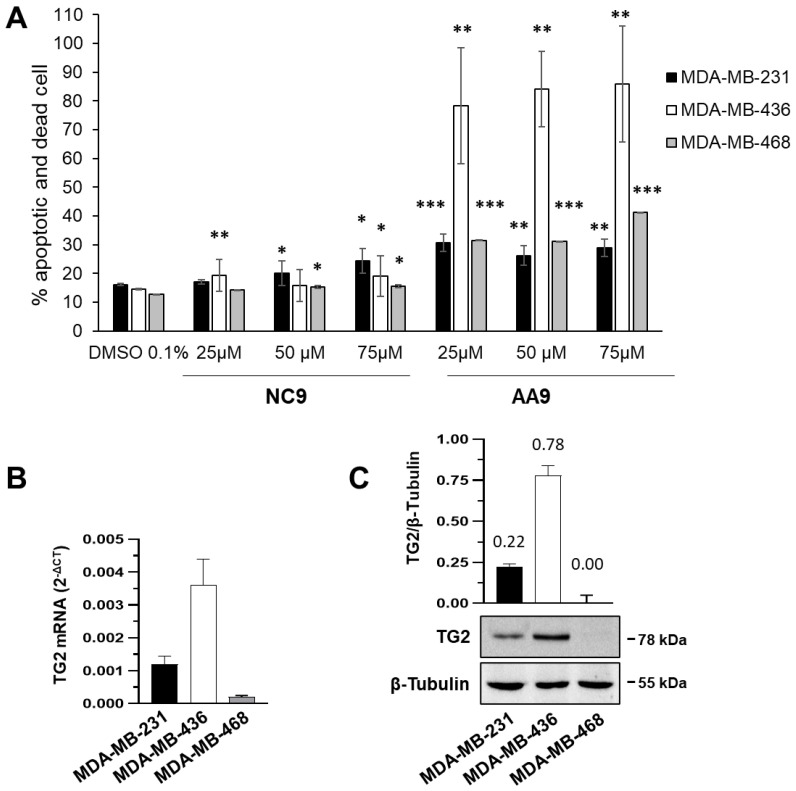
Apoptosis induced by TG2 inhibitors in BC cell lines (MDA-MB-231, MDA-MB-436 and MDA-MB-468). (**A**) Percentages of apoptotic and dead cells after treatment with AA9 and NC9 at the indicated concentrations. * *p* value < 0.05, ** *p* value < 0.01, *** *p* value < 0.001. (**B**) RT-qPCR analysis of TG2 mRNA basal levels in the 3 different cell lines cultured in control conditions. The levels of transcripts were determined using the 2^−ΔCT^ method and β-actin. Data of RT-qPCRs were analyzed on the 3 groups; we calculated *p* values by ANOVA; the *p* value threshold was < 0.01. (**C**) Representative Western blot analysis was performed with the indicated antibodies on cell lysates. TG2 expression was deduced from the chemiluminescence signal normalized with β-tubulin, taken as internal control for the equivalence of loaded proteins. All data are shown as the mean ± SD of three independent experiments. The original Western blots can be found in File S1.

To compare the effectiveness of two TG2 inhibitors as anticancer drugs, the novel AA9 and the well-known NC9, we evaluated apoptosis after 48 h of exposure in three BC cell lines, MDA-MB-231, MDA-MB-436 and MDA-MB-468, which all have triple-negative phenotype but are affected by distinct mutations. Both compounds induced apoptosis and cell death, as reported in Figure 1A. NC9 resulted in relatively low percentages of apoptotic/dead cells, even at 75 μM, for all three types of cells, as reported in the literature, when administrated alone [14,15,31,47,48,49,61]. AA9 produced greater effects, even when employed at lower concentrations, proving to be highly active in MDA-MB-436 cells. Interestingly, the latter expressed TG2 at a significantly higher level than the other cell lines, considering not only the accumulation of the transcript (Figure 1B) but also of the protein (Figure 1C), which was expressed at very low levels in MDA-MB-468 cells. 

These data show that AA9 induces a greater apoptotic effect than NC9, although this is not directly related to the amount of TG2 in the three different cell lines. 

### 3.2. The AA9 Inhibitor of TG2 Leads to a Significant Decrease in the Membrane Current

**Figure 2:**
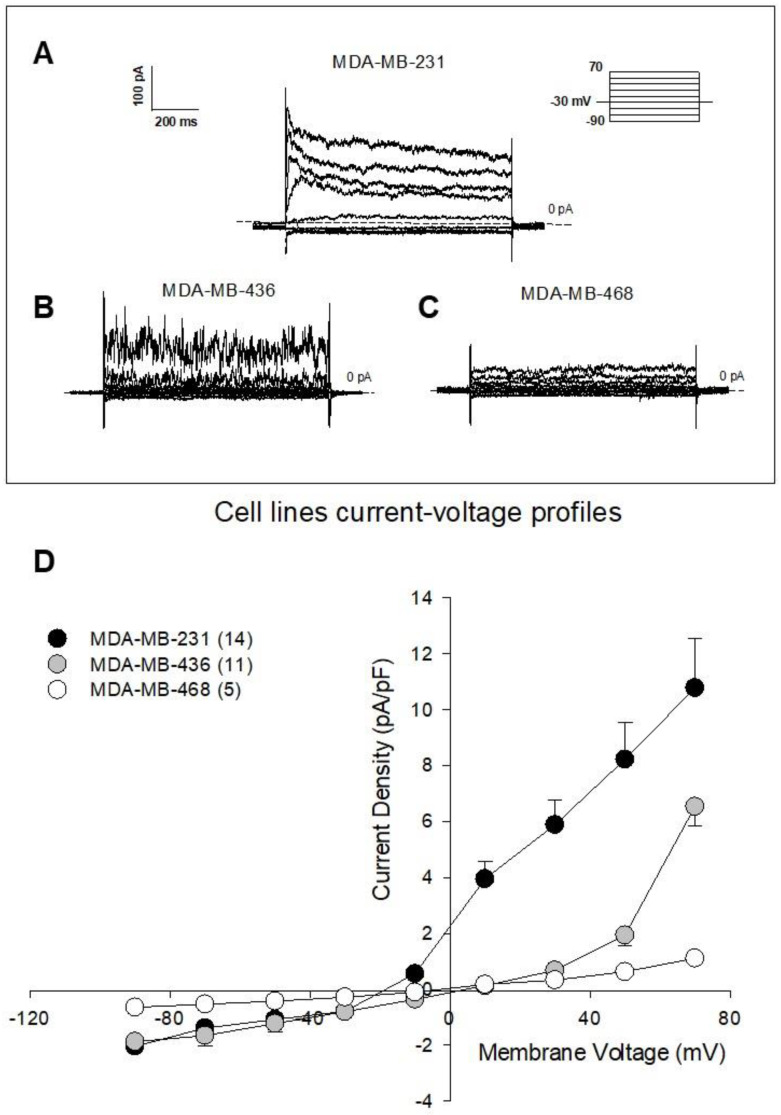
Membrane current in BC cell lines. Representative families of total recording currents in (**A**) MDA-MB-231, (**B**) MDA-MB-436 and (**C**) MDA-MB-468 cell lines. Dashed line indicates zero-current level. (**D**) Current-voltage relationship of MDA-MB-231 (*n* = 14), MDA-MB-436 (*n* = 11) and MDA-MB-468 (*n* = 5) cells. The results are expressed as average ± SEM. pA⋅pF^−1^ (picoAmpere/picoFarad).

To assay the intracellular effects of NC9 and AA9, we used the patch-clamp technique while recording the whole membrane current after injection of the compounds into a single cell. We applied a voltage protocol starting from −30 mV, which corresponds indicatively to their membrane potential, and ranging from −90 to +70 mV with 20 mV steps. Figure 2 displays representative families of total recorded currents in MDA-MB-231 (A), MDA-MB-436 (B) and MDA-MB-468 (C) cells, along with the current-voltage relationship (D). From their profiles, it emerges that MDA-MB-468 control cells did not display a significantly detectable voltage-dependent current, which was instead strongly evident in MDA-MB-231 cells, and albeit to a lesser extent, in MDA-MB-436 cells. 

**Figure 3:**
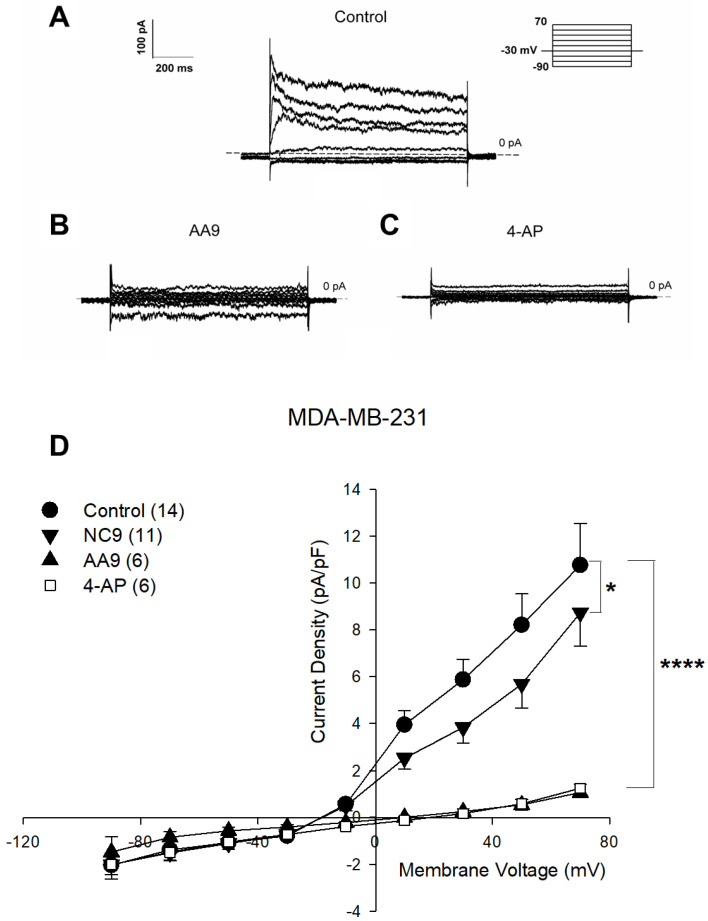
Representative families of total recording currents of MDA-MB-231 cells in (**A**) control conditions, (**B**) after treatment with AA9 and (**C**) after treatment with 4-AP. Dashed line indicates zero-current level. (**D**) Current-voltage relationship of control cells (*n* = 14), NC9 (*n* = 11), AA9 treated cells (*n* = 6) and 4-AP treated cells (*n* = 6). The results are expressed as average ± SEM; comparisons between curves were performed by two-way ANOVA: * = *p* < 0.05; **** = *p* < 0.0001. The results were expressed as average ± SEM. pA⋅pF^−1^ (picoAmpere/picoFarad).

For this reason, and following the minimal expression of TG2 in MDA-MB-468 cells, we carried out the following tests only in the last two cell lines. The recorded data, reported in Figure 3, show a significant decrease of 20% in the current density, caused by the injection of the reference compound NC9 into the MDA-MB-231 cells, yet the administration of AA9 was more effective than the administration of NC9, decreasing the current density by 90.1 ± 3.3% at the voltage of +70 mV (two-way ANOVA, *p* < 0.001). 

Moreover, the current reversal potential of control cells (E_rev_(CTL) = −17.9 ± 1.4 mV; *n* = 14) was significantly more negative than that of treated cells (E_rev_(AA9) = 12.7 ± 5.2 mV; *n* = 6; Student’s *t*; *p* < 0.0001), indicating the lower contribution of the K^+^ ions to the total current in the latter condition.

In order to demonstrate the involvement of the K^+^ voltage-dependent channels in MDA-MB-231 cells, we repeated the patch-clamp experiments while treating the cells with the specific inhibitor 4-AP [51,52]. After extracellular addition of 4-AP, we observed a reduction in the membrane current by 88.4 ± 3.5%, comparable to that obtained with AA9 (Figure 3D), so that the two curves seem to overlap.

**Figure 4:**
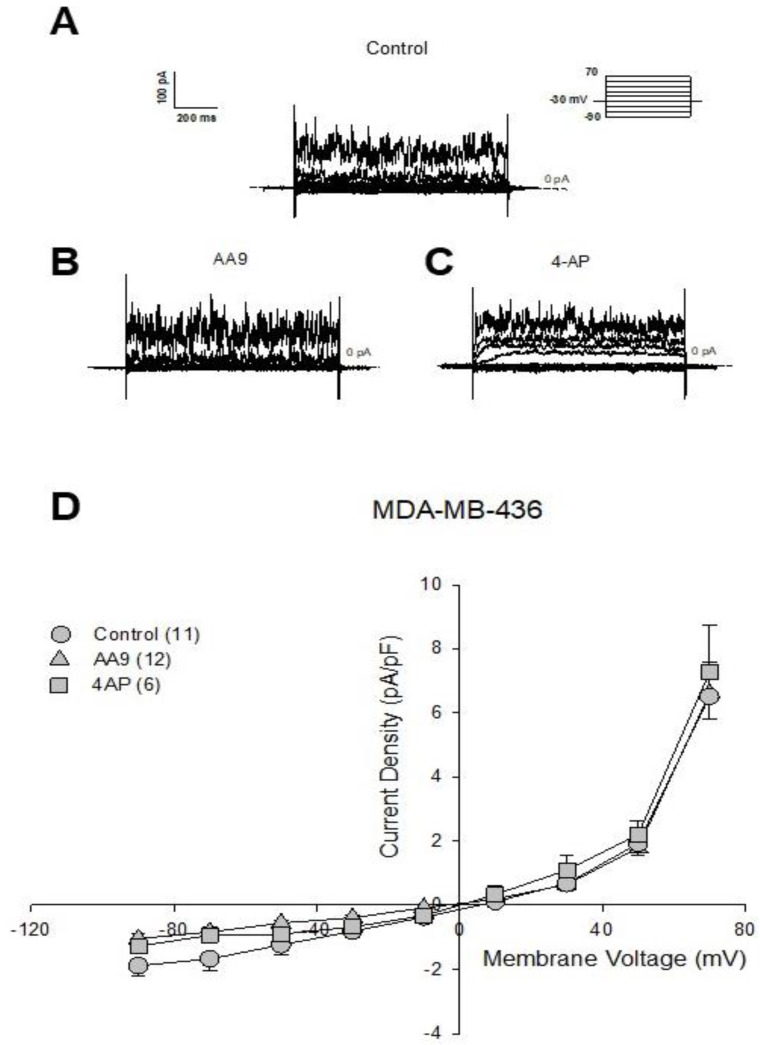
Representative families of total recording currents of MDA-MB-436 cells in (**A**) control condition, (**B**) treated with AA9 and (**C**) treated with 4-AP. Dashed line indicates zero-current level. (**D**) Current-voltage relationship of control cells (*n* = 11), AA9 treated cells (*n* = 12) and 4-AP treated cells (*n* = 6). The results are expressed as average ± SEM; comparisons between curves were performed by two-way ANOVA and were not significant. The results are expressed as average ± SEM. pA⋅pF^−1^ (picoAmpere/picoFarad).

We then investigated the effects of AA9 in MDA-MB-436 cells. The results, as shown in Figure 4, demonstrated that in this case, the TG2 inhibitor did not modify the current profile. 

Indeed, the current reversal potential of control cells (E_rev_(CTL) = 8.91 ± 2.7 mV; *n* = 11) and cells treated with AA9 (E_rev_(AA9) = −0.17 ± 7.6 mV; *n* = 12) were not significantly different (Student’s *t*; n.s.), nor was the value recorded for the cells treated with 4-AP different from the control (E_rev_(4-AP) = 5.5 ± 4.04 mV; *n* = 6), indicating the ineffectiveness of the inhibitors used in modifying the membrane current.

In conclusion, among the three tested triple-negative cell lines, only in MDA-MB-231 was the membrane current sensitive to the intracellular action of TG2 inhibitors in particular, that of AA9 in which ion fluxes strongly depend on voltage K^+^ channels, as confirmed by responsiveness to 4-AP as well.

### 3.3. Identification of the Voltage-Dependent Kv10.1 K^+^ Channel as an Interaction Partner of TG2

**Figure 5:**
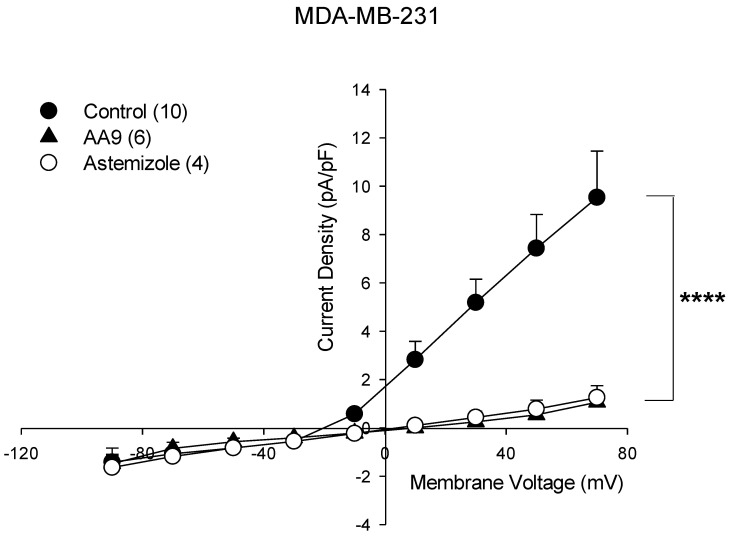
Current-voltage relationship of control cells (*n* = 10), AA9 treated cells (*n* = 6) and astemizole treated cells (*n* = 4). The results are expressed as average ± SEM; comparisons between curves were performed by two-way ANOVA (**** = *p* < 0.0001). The results are expressed as average ± SEM. pA⋅pF^−1^ (picoAmpere/picoFarad).

Our findings suggest interesting involvement of TG2 in the regulation of ion fluxes. Focusing on the identification of K^+^ channels as a possible target of TG2, the literature suggests that one of the most abundant in cancer cells is the voltage-dependent Kv10.1. We propose that the Kv10.1 channel could be involved in this relationship, particularly because it sustains the proliferation [62] and migration of MDA-MB-231 cells [53]. Thus, we performed similar path-clamp experiments using astemizole (at 10 μM concentration), which is a selective inhibitor of the Kv10.1 channel. As shown in Figure 5, the current profile overlaps with that obtained in the presence of AA9, suggesting that this specific channel could be a possible partner of TG2. 

To assess their physical interaction, we investigated the association by the use of specific antibodies.

Considering that other voltage-dependent channels, such as Kv11.1, are involved in EMT and metastasis dissemination in MDA-MB-231 cells [63], we cannot exclude interactions of TG2 with the Kv11.1 channel, which is very similar to Kv10.1 but shows opposite activity. Indeed, in xenograft tumors derived from MDA-MB-231 cells, Kv11.1 activation reduced the size of the tumors and metastasis [63]. Kv10.1 and Kv11.1 belong to the same family and are very similar and difficult to distinguish from each other, because of the high analogy between them and with other members of the family [42]. Thus, we have chosen an antibody directed against the C-terminus (aa 751–875) of the Kv10.1 and one directed against the N-terminus (aa 96–270) of Kv11.1, after verification on the NCBI website that the protein sequences of the epitope recognized by the chosen antibodies look very different in these regions (https://blast.ncbi.nlm.nih.gov/Blast.cgi?PROGRAM=blastp&PAGE_TYPE=BlastSearch&LINK_LOC=blasthome, accessed on 30 August 2022). 

**Figure 6:**
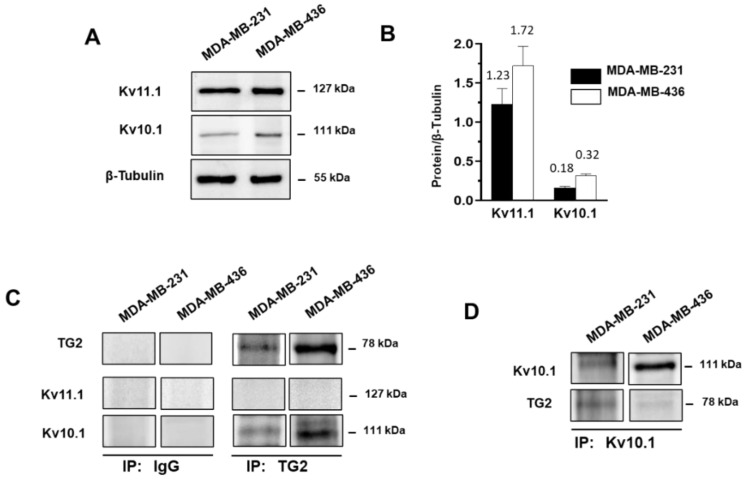
Analysis of the association between TG2 and K^+^ channels. (**A**) Representative immunoblot analysis was performed with the indicated antibodies on lysates from MDA-MB-231 and MDA-MB-436 cells in control conditions. (**B**) Levels of Kv10.1 and Kv11.1 were obtained from chemiluminescence analysis and normalized with respect to β-tubulin. (**C**,**D**) Representative Western blot analysis with the indicated antibodies in immunoprecipitates (IP) with antibodies directed against TG2, Kv10.1 or a non-specific IgG.

Before setting up immunoprecipitation (IP) conditions, we checked the presence of both channels by Western blot using specific antibodies, and we verified their expression in MDA-MB-436, and in MDA-MB-231 cells (Figure 6A,B). Since TG2 was slightly appreciable by Western blot in the MDA-MB-468 cells (as previously reported in Figure 1C), this cell line was excluded by IP experiments. 

To explore the association between TG2 and one of these K^+^ channels, we first employed an antibody against TG2 to immunoprecipitate the complexes containing the enzyme from cellular lysates. Figure 6C shows that in both cell lines only the Kv10.1 was recovered from the formed complexes. In addition, inverse assays carried out IP with antibodies against Kv10.1 demonstrated the presence of TG2 in the immunoprecipitates from both cell lines (Figure 6D). These data allowed us to assess the interaction of TG2 with Kv10.1 in MDA-MB-231, in which we revealed an effect of TG2 inhibition on membrane current, but also in MDA-MB-436, in which TG2/Kv10.1 association cannot be correlated with ion flux (Figure 6C).

### 3.4. Analysis of the Metabolites Secreted after AA9 Treatment to Find Cell-Response Markers 

The role of membrane depolarization in association with secretion processes has long been known [64]. It has been associated with hormone extrusion, as studied in rat cerebrocortical cells [65], and has been extensively described in pancreatic islets, in which insulin secretion depends on K^+^ channel activity [66,67]. 

To identify relevant markers for cell-sensitivity to AA9 treatment, we focused on secreted metabolites. Thus, we performed ^1^H-NMR-based metabolic profiling of the supernatants to investigate the impacts of AA9 on metabolites exchanged between MDA-MB-231 cells and culture medium; as a further control, we assessed the metabolic content of the fresh medium (Figure S1). We identified 40 metabolites in the supernatants of cells cultured in the presence and absence of the TG2 inhibitor and 36 in the fresh medium. Most of the metabolites were consumed by the cells, but the following were secreted into the medium: alanine, pyruvate, 2-hydroxybutyrate, 2-oxoisocaproate, 3-methyl-2-oxovalerate, dimethylamine and methylamine and urea.

**Figure 7:**
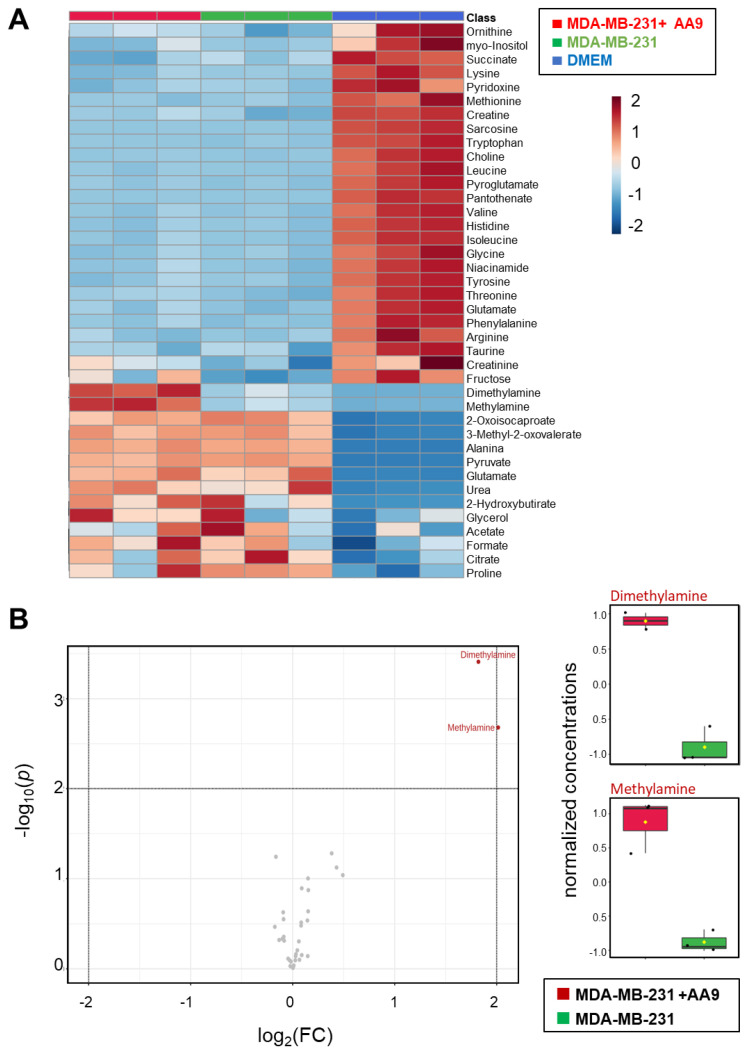
Metabolomics analysis of culture supernatants. (**A**) Heatmap of the metabolites. Illustration of the metabolite level datasets for fresh medium and supernatants of untreated and MDA-MB-231 cells treated with AA9. The rows indicate the identified metabolites, whereas the columns indicate the different triplicate samples for each group. Red and blue represent higher and lower concentrations of metabolites, respectively. The heatmap scale ranges from −2 to 2 on a log2 scale. (**B**) Univariate statistical analysis. On the left, volcano plot shows the univariate statistical analysis of extracellular metabolomes obtained from MDA-MB-231 cells treated with AA9 versus control; on the right are evidenced the significantly dysregulated metabolites. The analysis was based on |FC| > 2 and *p* values < 0.01. Red and grey dots represent up-regulated and not significantly changed metabolites, respectively.

Figure 7A depicts the correlation heatmap of the whole set of samples, showing the relative abundance of the identified metabolites. The heatmap reveals that samples are clustered according to the defined groups. The fresh medium is clearly different when compared with MDA-MB-231 supernatants, a consequence of the exchange of metabolites. However, differences were observed between AA9-treated and untreated MDA-MB-231 cells that exhibited distinct metabolite patterns. To identify the metabolites that discriminate between AA9-treated and untreated cells, we performed a univariate statistical analysis comparing the metabolite profiles of the two sample groups. The volcano plot with *p* < 0.01 and fold change |FC| > 2 as thresholds for significance is shown in Figure 7B. The metabolites discriminating between the two groups were methylamine and dimethylamine; both were found at a higher concentration in the supernatants 16 h after AA9 administration.

## 4. Discussion

Our first goal was to verify the efficacy of the novel TG2 inhibitor AA9 by comparison with the well-known NC9, in three triple-negative BC cells. AA9 provided a general apoptosis/death effect on the selected cell lines, which, however, did not directly correlate with the level of TG2. Due to the multifunctionality of this enzyme, it should be emphasized that it is difficult to attribute apoptosis induction to a specific TG2 function, owing to the roles displayed inside and outside the cell. The situation is further complicated by the presence of altered variants [31,68,69,70], whose functions are still a matter of investigation. 

An extracellular role of TG2 contained in vesicles has been recently demonstrated on L-type calcium channels [36], localized on nerve terminals in the pre- and postsynaptic parts of the central nervous system. The involved voltage-gated calcium channels associated with several functions in cancer are, however, downregulated in BC [71]. 

To distinguish the relevance of TG2 inside the cell, excluding interference by extracellular functions of the enzyme, we employed the patch-clamp technique, which allowed us to scrutinize the changes induced by TG2 inhibitors, creating a continuous solution between cytosol and the contents of the internal space of the micropipette. In this manner we analyzed changes following intracellular effects and observed prompt current modifications.

We tested the TG2 inhibitors in three BC cell lines, and immediately their current profiles showed that not all the cell lines were suitable, owing to the lack of significant voltage-dependent currents. Therefore, we restricted the treatments only to the MDA-MB-436 and MDA-MB-231 cells, the latter being the most responsive. In fact, the measurements demonstrated that TG2 inhibition leads to a significant decrease in membrane current only in MDA-MB-231 cells, which was more pronounced with AA9 than with NC9. The similar trend of the current-voltage relationship after AA9 and 4-AP treatments of the cells suggested the involvement of voltage-dependent K^+^ channels.

A link of inter-functionality between the latter and TG2 has been described in the β cells of the Langerhans islets, where membrane depolarization drives the insulin secretion [66,72]. The variations in the intracellular Ca^2+^ concentration triggered by changes in K^+^ fluxes activate TG2 [73], leading TG2 to interact with actin filaments and cytoskeleton structure, resulting in driving granules out of the cell [74]. 

Instead, another type of relation, reported in vascular smooth muscle cells, found TG2 modulating conductance of the Maxi-K^+^ channels [43]. In our BC cells 4-AP did not modify the current profiles associated with channels of this kind, as already reported [51,53].

The influence of TG2 on K^+^ flux has already been studied through specific inhibitors promoting the closed conformation of the enzyme. This event seems to regulate the relaxed state of arteries lowering blood pressure [25]. These effects take place following the interaction between TG2 and another voltage-dependent channel, Kv7, modulated by the G-protein function of TG2, as demonstrated using the specific inhibitor LDN 27129 [44]. However, Kv7 channels do not seem to have any important role in eliciting the membrane current of BC cells [40], whereas Kv10.1 is involved [75]. The expression of the Kv10.1 channel, as well as that of Orai1, which sustains Ca^2+^ influx, depends on the activation of the discoidin domain receptor 1 pathway, which is mediated by collagen type I and activated via ERK1/2 [76,77]. 

Considering that Kv10.1 is one of the most relevant channels associated with cell division, EMT and metastasis and for this reason is a good candidate for therapy [78], we tried to define its possible interplay with TG2. The use of the specific Kv10.1 channel inhibitor astemizole confirmed our hypothesis, since its effects on the membrane current overlap those of the TG2 inhibitor AA9. We excluded the possibility that it depends on Ca^2+^ current, because this was travelling inward for the entire voltage range we explored; indeed, the reversal potential of the Ca^2+^ current is much higher than the tested +70 mV, and furthermore, it inactivates before reaching it [79]. 

By use of co-immunoprecipitation, we demonstrated in vitro the formation of complexes between Kv10.1 and TG2, validating the possibility that their physical interaction is at the basis of the role of TG2 in K^+^ fluxes. Despite the inhibitor AA9 blocking both transamidation and GTP-binding functions of TG2 [35], we believe that the effects on Kv10.1 can be attributed to the latter, since TG2 regulates the Kv7 channel by G-protein activity [44]. However, we cannot exclude further intracellular interferences of TG2, given its ability to modify the cytoskeleton, another process closely related to the migration and survival of BC cells [14]. 

Regarding the metabolic analysis we carried out on supernatants, a significant increase in both dimethylamine and methylamine was detected 16 h after the addition of AA9, making them possible markers of drug sensitivity. Dimethylamine and methylamine could constitute further factors in implementing the crosstalk TG2/K^+^ channel. An effect of amines on TG2 has been described as associated with insulin secretion by pancreatic islets [80], which showed different behavior for trimethyl-, dimethyl- and methylamine. While trimethylamine did not show any effects, dimethylamine and methylamine impaired the release of insulin in a way that is not necessarily directly associated with the transamidation activity of TG2. This effect was attributed to earlier changes impairing permeability to K^+^ ions, and decreased conductance and Ca^2+^ uptake are consequences of a higher K^+^ extracellular concentration [81].

The increases in secreted dimethylamine and methylamine could depend on the effects of TG2 inhibition on other targets. Notoriously, TG2 forms complexes with PLCδ1, thereby inhibiting it, whereas the GTP binding by TG2 allows PLCδ1 release and activation [82,83,84]. In this manner, the G-protein function of TG2 modulates PLCδ1, which catalyzes the degradation of membrane phospholipids, interfering not only with the inositol pathway, but also with the metabolism of phosphatidylcholine, one of the possible substrates of phospholipase [85]. PLCδ1 is one of the key enzymes in triple-negative BC cells [86], where it catalyzes the degradation of phosphatidylcholine into 1,2-diacylglycerol and phosphocholine, which is then converted into choline. In humans, dietary choline is mainly degraded by intestinal microbacteria into trimethylamine [87] and then into dimethylamine [88], but these reactions have not been extensively investigated in mammals, so it remains unclear if there is evidence of the existence of an endogenous pathway [89].

It might be important to underline also that the binding of PLCδ1 by TG2 supports autophagosome degradation in MDA-MB-231 cells [90]; therefore, blocking the formation of this complex could have therapeutic implications. 

Considering potential therapeutic strategies, it could be interesting to combine the use of AA9 with inhibitors of the Kv10.1 channel, known to regulate cell cycle [61], drug resistance and tumor progression [91], some of which have already been proposed for clinical applications [78]. In particular, it will be interesting to analyze the effectiveness of the potential synergistic action of TG2 inhibitors and astemizole which are known to work in combination with other drugs [92,93] which in this case would also have the same target, the Kv10.1 channel [94].

A synergy of TG2 plus Kv10.1 inhibitors could be effective, especially in those BC types presenting mutations leading to overexpression of the K^+^ channel, such as TP53 in MDA-MB-231 [95]. This would represent a potential treatment of more aggressive BCs, which often show upregulation of TG2 expression [31,96], and identify AA9 as a good adjuvant candidate. However, with the goal of clinical application, we must not forget that TG2, despite its dysregulation in cancer, has ubiquitous tissue distribution in normal cells, so it will be important to direct inhibition against specific targets exclusive to cancer cells, in order to develop delivery systems aimed at obtaining an effective selective treatment. Non-specific effects could be overcome using nanobodies, as in the administration of Kv10.1 blockers, improving the selectivity for specific tumor cells [97].

## 5. Conclusions

Using a multidisciplinary approach, we investigated the association of TG2 with K^+^-voltage dependent channels in three triple-negative cell lines with different features, demonstrating interference of TG2 mainly with Kv10.1. Of note, the use of AA9 to inhibit TG2 demonstrated that it has selective effectiveness, so it could be helpful especially in combined therapy of cancers with specific phenotypic/genotypic characteristics.

## Data Availability

All data used to support the conclusions are included in the manuscript.

## Supplementary Materials

The following supporting information can be downloaded at: https://www.mdpi.com/article/10.3390/cancers15010178/s1. Figure S1: Multicomponent analysis and VIP (variable importance in projection) score of supernatants derived from MDA-MB-231 cells untreated and treated with AA9. (**A**) PLS-DA 2D plot model showing separation between the metabolic composition of the supernatants of MDA-MB-231 cell cultures in the absence and presence of AA9 and of the fresh DMEM medium. (**B**) Metabolite ranking (top 15 metabolites) according to the VIP scores, resulting from the separation by component 1 in the PLS-DA scores plot. Higher VIP scores indicate a more relevant contribution to discriminating between groups. The colored boxes on the right side of the VIP score plot denote the relative metabolite abundance in each cluster of the PLS-DA plot. File S1: The original Western blots.
