# Supplementary material for: A Multidisciplinary Approach Establishes a Link between Transglutaminase 2 and the Kv10.1 Voltage-Dependent K+ Channel in Breast Cancer
Source: Cancers (Basel). 2022 Dec 28;15(1):178. doi: 10.3390/cancers15010178 (PMC9818547; doi:10.3390/cancers15010178)
Supplement: Supplementary file 1 [file cancers-15-00178-s001.zip › cancers-2015064-supplementary/File S1 - The original western blots.pdf]

File S1

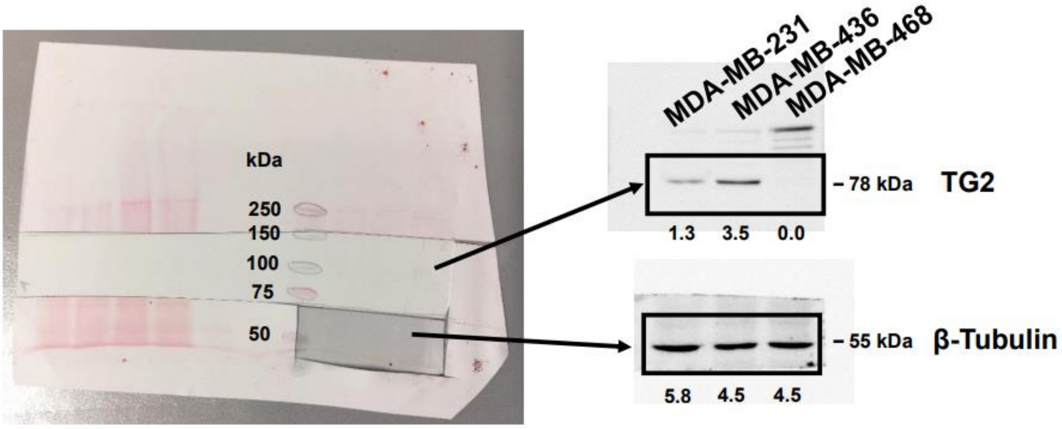

Figure S2. The original western blots of Figure 1C.

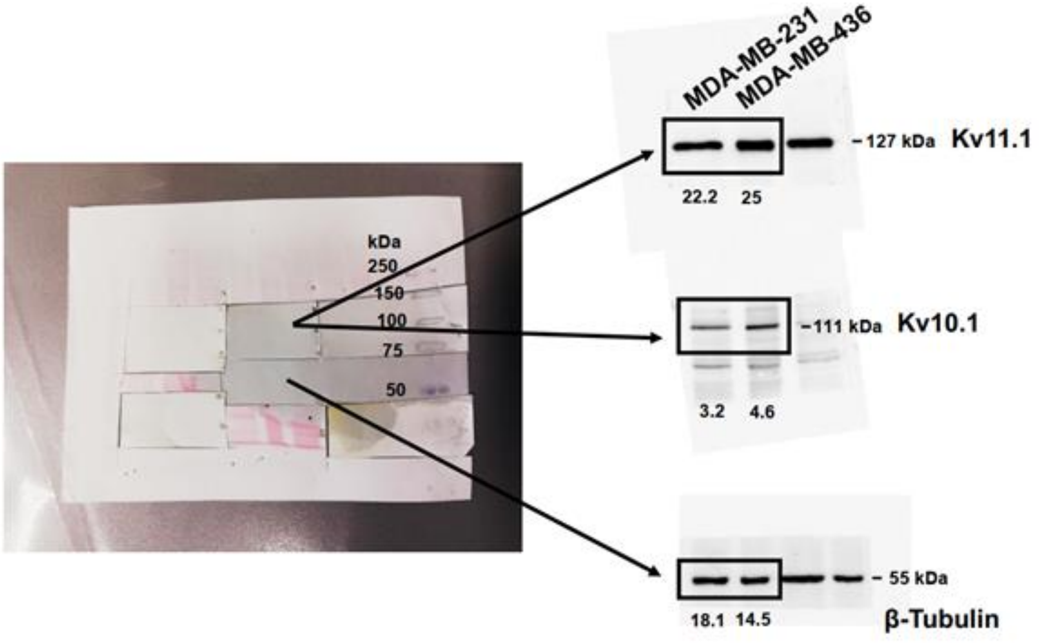

Figure S3. The original western blots of Figure 5A.

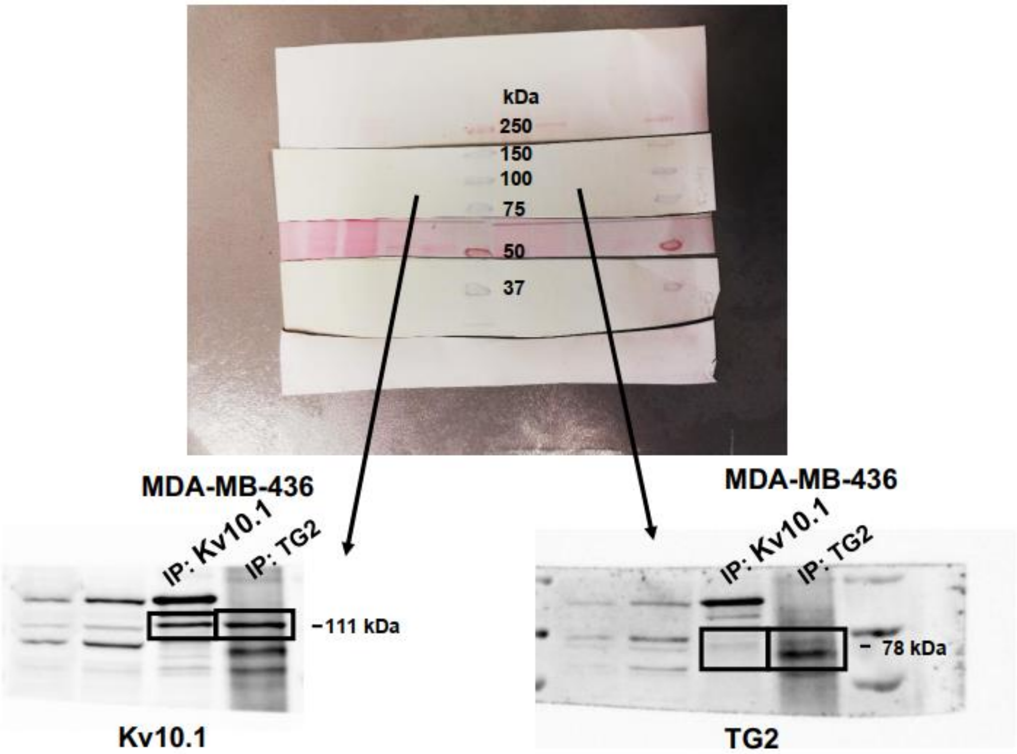
kDa
250
150
100
75
50
37
MDA-MB-436
IP: Kv10.1
IP: TG2
–111 kDa
Kv10.1
MDA-MB-436
IP: Kv10.1
IP: TG2
– 78 kDa
TG2

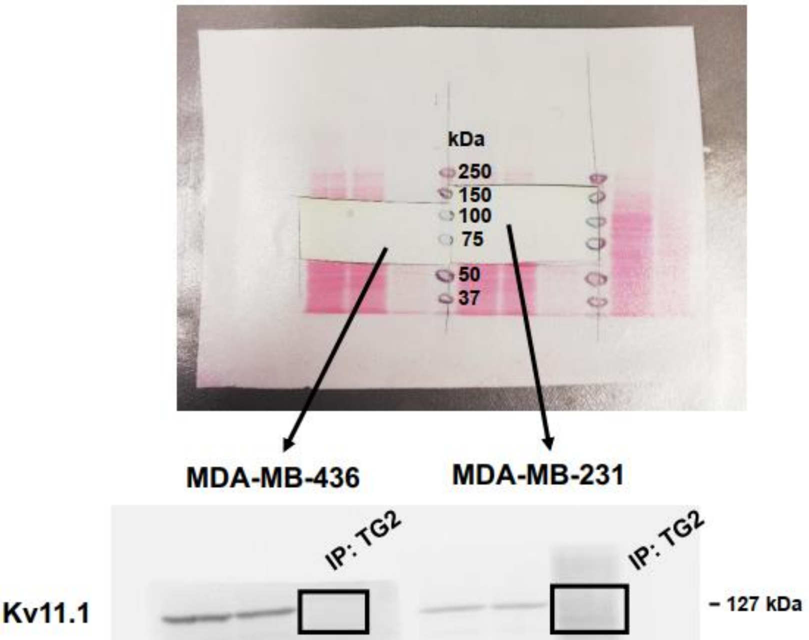
kDa
250
150
100
75
50
37
MDA-MB-436
MDA-MB-231
IP: TG2
IP: TG2
Kv11.1
– 127 kDa

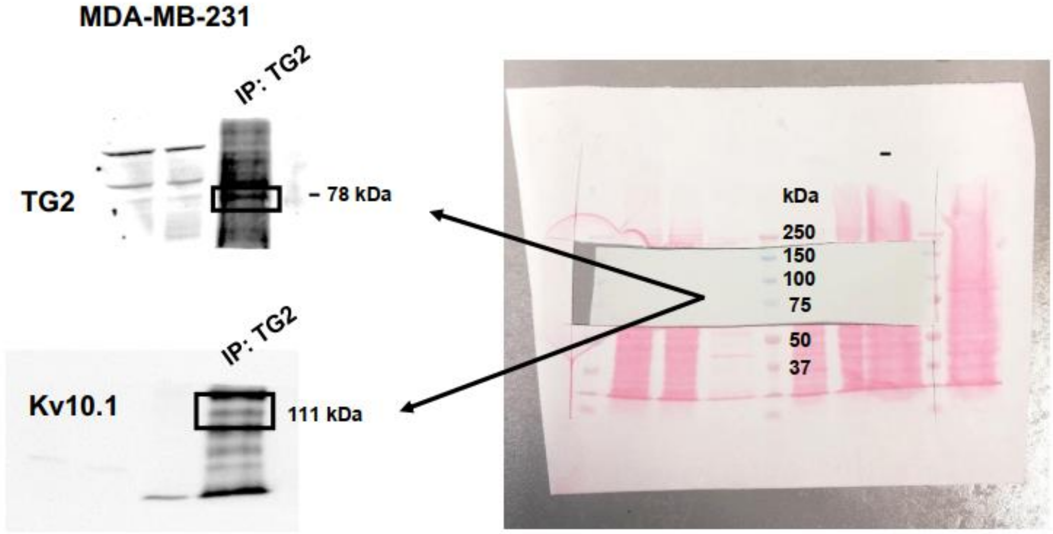
MDA-MB-231
IP: TG2
TG2
– 78 kDa
IP: TG2
Kv10.1
111 kDa
kDa
250
150
100
75
50
37

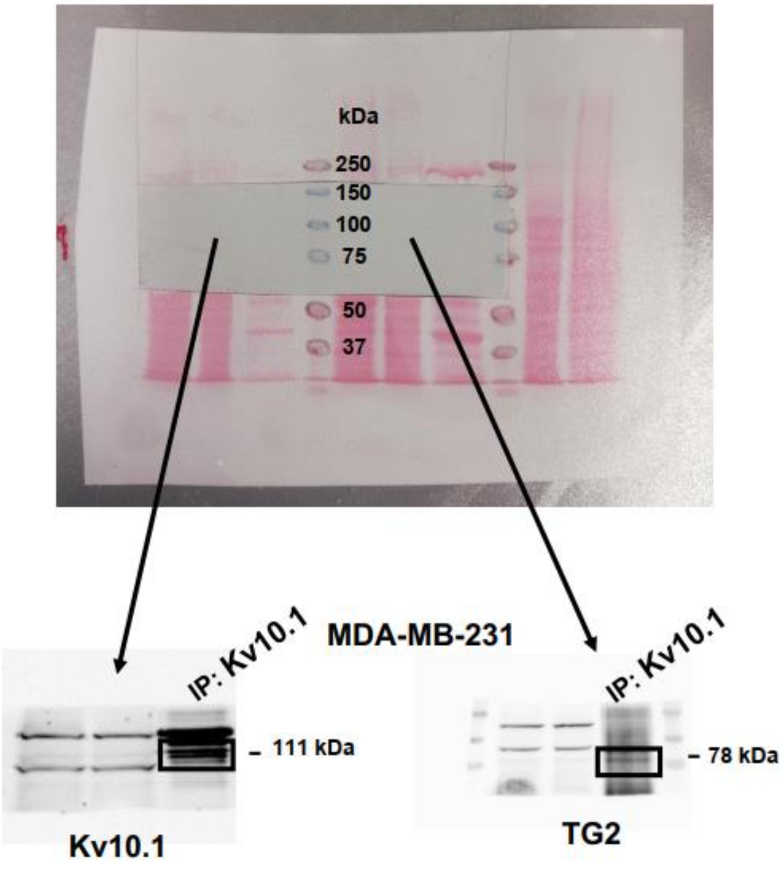
kDa
250
150
100
75
50
37
IP: Kv10.1
MDA-MB-231
IP: Kv10.1
– 111 kDa
– 78 kDa
Kv10.1
TG2

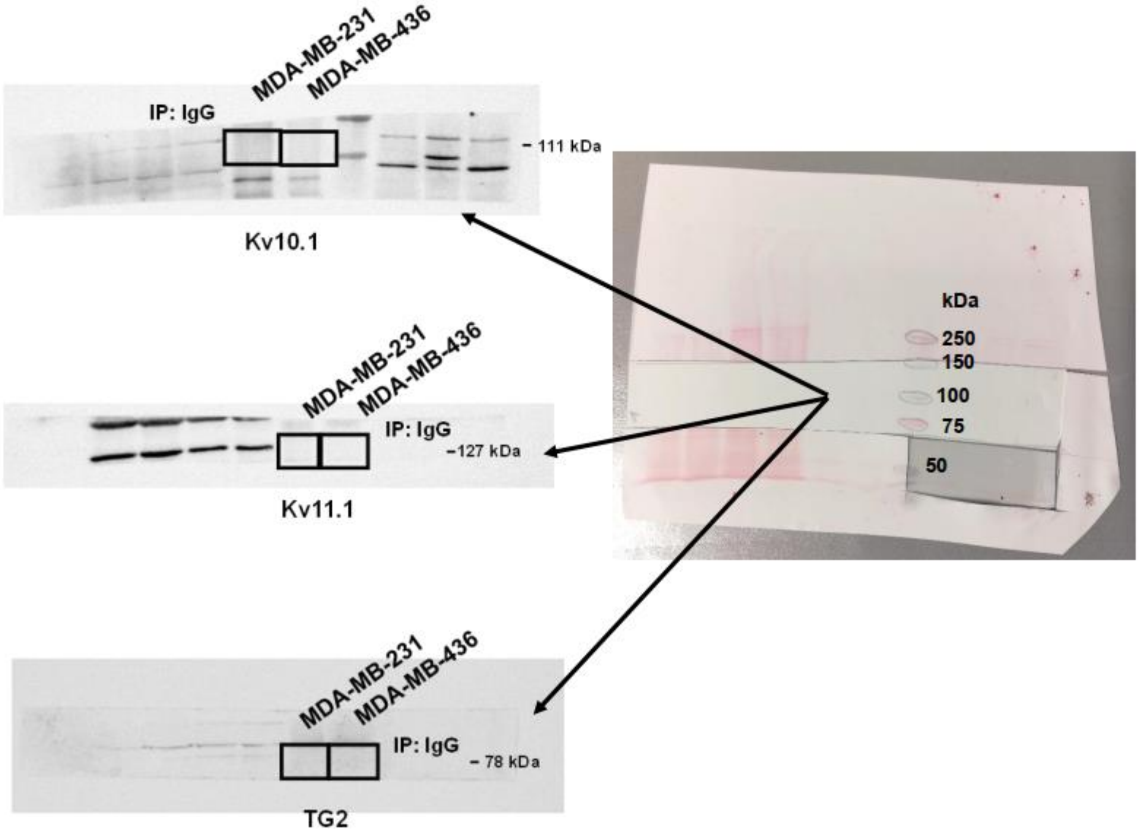

Figure S4. The original western blots of Figure 5C and Figure 5D.
