# Supplementary figures and images for: A Multidisciplinary Approach Establishes a Link between Transglutaminase 2 and the Kv10.1 Voltage-Dependent K+ Channel in Breast Cancer
Source: Cancers (Basel). 2022 Dec 28;15(1):178. doi: 10.3390/cancers15010178 (PMC9818547; doi:10.3390/cancers15010178)

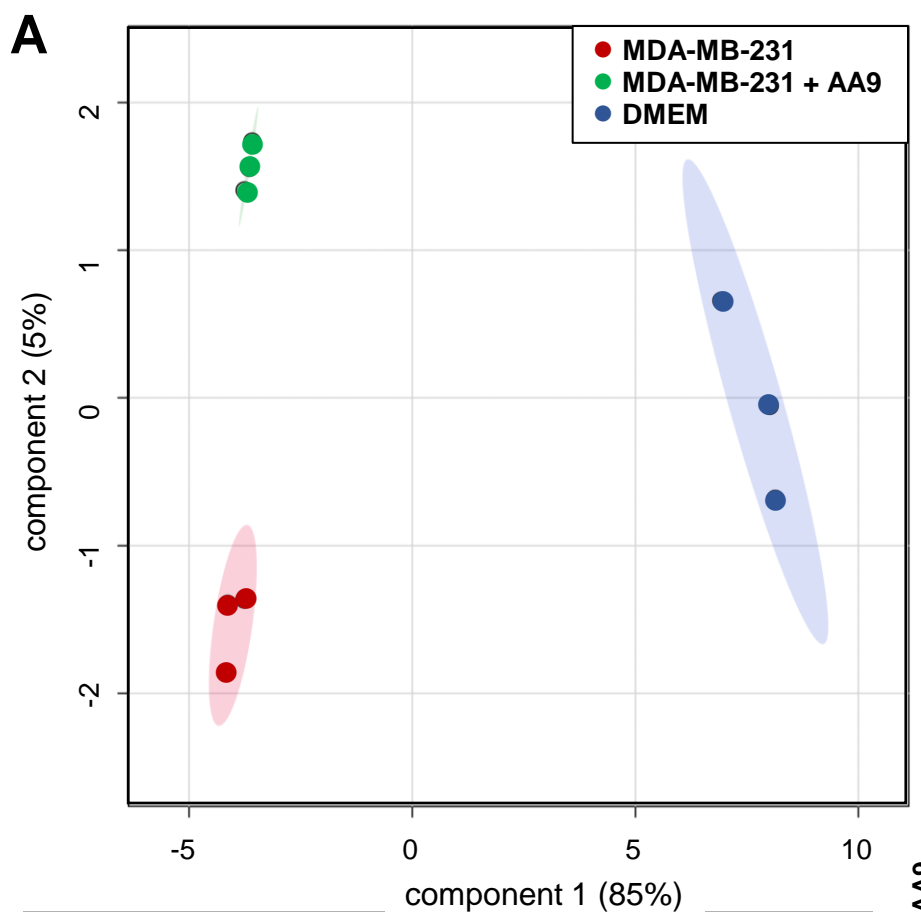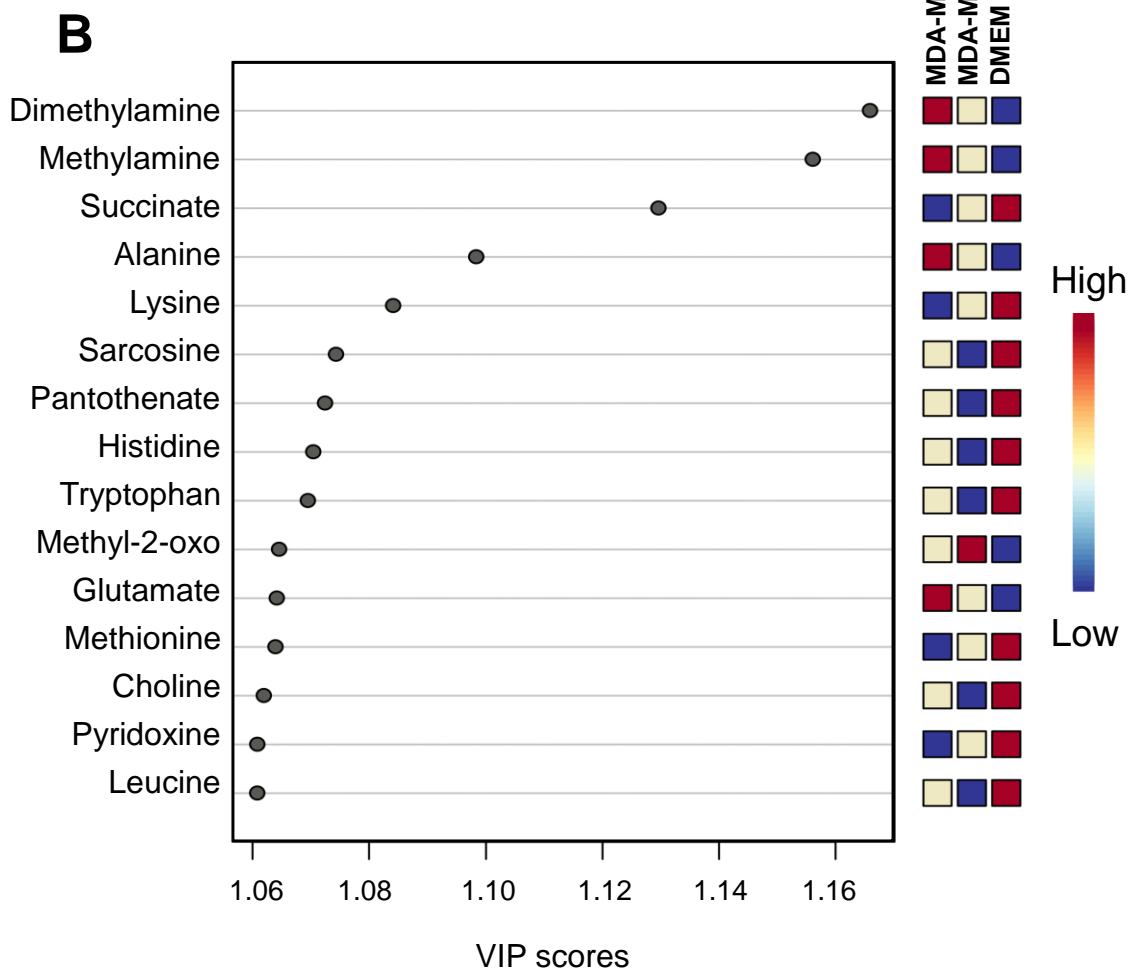

Supplement: Supplementary file 1 [file cancers-15-00178-s001.zip › cancers-2015064-supplementary/Figure S1.pdf]
